# Spatiotemporal Frameworks for Infectious Disease Diffusion and Epidemiology

**DOI:** 10.3390/ijerph13121261

**Published:** 2016-12-20

**Authors:** Peter Congdon

**Affiliations:** School of Geography and Life Sciences Institute, Queen Mary University of London, Mile End, London E1 4NS, UK; p.congdon@qmul.ac.uk; Tel.: +44-207-882-8200

## 1. Background

Emerging infectious diseases, and the resurgence of previously controlled infectious disease (e.g., malaria, tuberculosis), are a major focus for public health concern, as well as providing challenges for establishing aetiology and transmission. Novel aspects affecting spread of infectious disease are factors such as globalisation, population growth, environmental change and antibiotic resistance [1].

Understanding the diffusion of infectious disease at population level in this context is of major importance in managing outbreaks of new or resurgent infectious disease. Infectious disease diffusion through space and time is an example of a broader class of spatiotemporal diffusion processes, involving movement across space and changing extent through time [2,3].

## 2. Diffusion Processes

With regard to infectious disease spread, spatiotemporal diffusion has been considered both for historic diseases, such as bubonic plague [4], and for recent infectious disease outbreaks such as the H1N1 pandemic, recurrent epidemics of dengue fever, and the spread of ebola [5]. Infectious disease spread among animals is subject to similar considerations [6]; up to a half of human infectious diseases have a zoonotic origin, that is, are transmitted from animals, with a higher proportion among emerging infectious diseases [7].

In the past, infectious disease diffusion was based primarily on contagious spread of the disease, but increasingly disease can spread quickly via transport links at regional and global scale, a process known as network diffusion [8]. Other principles may be paramount such as hierarchical spread from large cities to smaller towns, while impediments to spread (e.g., distance decay effects) are also relevant.

One may also consider co-dependence between infectious diffusion and other outcomes, such as malformation in newborns linked to zika virus infection in mothers. For example, [9] consider the dynamics of HIV sero-discordancy (one partner testing HIV seropositive, while the other testing HIV seronegative), and of sero-concordancy, as against HIV prevalence itself.

## 3. Analytic Methods

Analytic tools are of obvious importance in measuring and analysing disease diffusion. An established tradition in epidemiological research has been to use surveillance and spatiotemporal visualisation techniques. These are used to identify the spread of infectious disease outbreaks, provide early warnings [10], identify the source of infection [11], predict the eventual disease extent [12], or permit intervention to prevent further spread of the disease.

Geographic information system (GIS) methods and GIS-based simulation in particular have utility in describing and analysing spatiotemporal infectious disease patterns [13,14]. For example, [15] describes DengueME, an open source platform to simulate dengue disease, implemented over a GIS database. DengueME represents Aedes aegypti population dynamics, human demography and mobility, urban landscape and dengue transmission mediated by human and mosquito encounters. Data mining applied to internet search and social media data is being increasingly applied [16].

A wide range of sophisticated mathematical and statistical techniques [17,18] have also been developed. These include measuring spatial and space-time clustering [19,20] to detect existing high risk areas or areas at risk of disease influx, while mathematical diffusion and epidemic models [21,22] focus especially on the time pattern of disease spread.

Incorporating spatial dependence for analysis at large scale geographies is important since environmental exposures, such as climate and land use, and other factors affecting transmission such as antibiotic resistance [14] and socio-economic status, are spatially dependent. So analyses that do not account for spatial dependence in exposures may overstate significance effects, whereas analysis allowing for spatial dependence will provide a better fit [23].

Illustrating application of such methods, Dong et al. [24] develop a predictive risk map for the distribution of the HN79 outbreak in China in 2013–2014, with spatial–temporal autocorrelation incorporated into logistic regression analysis along with environmental and climatic risk factors. By contrast, time dependence is the focus in the hybrid approach of Zhou et al. [25], combining autoregressive integrated moving average (ARIMA) and nonlinear autoregressive neural network (NARNN) models to forecast the prevalence of schistosomiasis. Li et al. [26] use space and space–time scan statistics and trajectory similarity analysis to establish differences in the location and evolution of new and retreated smear-positive TB patients. Adegboye and Adegboye [27] apply a dynamic transmission multivariate time series model to cutaneous leishmaniasis in Afghanistan, e.g., see [28], to evaluate the effects of three environmental layers as well as seasonality in the data. Furthermore, ecological niche modeling was used to study the geographically suitable conditions for cutaneous leishmaniasis using temperature, precipitation and altitude as environmental layers.

## 4. Infectious Disease Aetiology and Disease Spread

Underlying and modulating space–time patterns in infectious diffusion are a variety of environmental and socio-economic factors, including population density and growth [7], and changing transport patterns [29]. For example, changes in the incidence of dengue fever in tropical countries have been related both to urbanization, increased air travel, and climatic factors such as rainfall, temperature, and humidity [30]. Health care access and effectiveness, vaccination uptake, and drug availability, especially in developing countries, also affect infectious disease control [31,32].

As illustrative applications, Zhang et al. [33] analyse the distribution of bacillary dysentery across China in terms of seasonality and meteorological factors, urban–rural disparities, and distribution of Shigella species. They find links to economic development and identify endemically high-risk regions in western China. Cao et al. [34] identify a similar spatial pattern for TB incidence in Chinese provinces and find links to climatic variables. Moise et al. [35] find that impacts on malaria incidence of seasonality and elevation may be modified by high population density and economic activity patterns (such as intensive subsistence farming practice) which affect exposure. Xu et al. [36] develop an ecological niche model for the spatial distribution of H7N9 cases using environmental, climatic and anthropogenic variables (distribution of live poultry processing factories, farms, and human population density), and find the latter as having greatest predictive value. Regarding the impact of health care access, a study of care-seeking for diarrhoea in Southern Malawi [37] show obstacles to obtaining healthcare advice, such as distance and transport costs to health facilities, as well as prolonged waiting times.

In a longer time perspective, environmental factors such as climate change [38], air pollution [39], ecosystem disruption and the loss of biodiversity are also important for understanding and controlling infectious disease. For example, deforestation has been linked to changing patterns of malaria and schistosomiasis. Lal [23] considers impacts of climate change on zoonotic transmission of cryptosporidiosis, as the parasite is easily transmitted through the environment, particularly through water. Historical time-series modelling of cryptosporidiosis incidence with rainfall has reported positive associations in a global meta-analysis of cryptosporidiosis seasonality [40]. Padilla et al. [41] consider impacts on infant mortality (often linked to infection) of socioeconomic factors and air pollution.
